# Virtual diversity revisited

**DOI:** 10.1177/03063127251330545

**Published:** 2025-04-11

**Authors:** Harry Collins, Robert Evans, Luis Reyes-Galindo

**Affiliations:** 1 Cardiff University, Cardiff, UK; 2 Independent scholar

We thank Laurent (2025), Parthasarathy (2025), Subramaniam (2025), and Thorpe (2025) for putting so much work into criticizing the article (Collins et al., 2025) we’ll refer to as ‘*VDiversity*’. We were allowed about 3,500 words to respond to the more than 13,000 words of ‘*VCritique*’, the four critical comments, and this means that we must deal mostly in generalities.^1^

The movie, *Don’t Look Up,* satirizes the collective failure to address the threat posed by climate change with the reaction to the impending destruction of terrestrial life by a comet. The response to the comet is ‘Don’t look up’. For a long time, STS scholars seemed determined not to look up, insisting that they had no responsibility for the comet of Trumpist populism. Thus, Sismondo (2017, p. 588) is ‘hard-pressed to see much in common between … the post-truth era and … STS’; and Lynch (2017, p. 597) says ‘it is the height of hubris to suggest that our field gave rise to, or is otherwise responsible for, the rhetorical means through which controversies have been ‘manufactured’’. *VCritique* indicates that STS may, belatedly, be raising its head.

We and these critics have at least six things in common, one in-between item, and three clear disagreements:

(1) We all want to safeguard expertise against the predations of the new US government (which determines all our fates) and we all think STS has a role.
(2) We all think the profession of science should be more diverse. For Collins, Evans, and Reyes-Galindo, this is a matter of social justice and applies equally to any profession.
(3) We agree virtual diversity would be a good thing if it could really work.
(4) We agree that successful virtual diversity will be hard to accomplish.
(5) We agree that policymaking is the job of policymakers while scientists should only offer advice.
(6) We agree that science in practice is invested with values. Collins, Evans and Reyes-Galindo understand science as ‘craftwork with integrity’, with part of the craft being the right choice of epistemic and social values (Collins, 2023; Collins & Evans, 2017). The role of values is most obvious when the science intersects with policymaking (Collins & Evans, forthcoming).
(7) We partially agree about the meaning of local case studies. The most closely worked out example in *VCritique* is Flint River pollution, which is used by Parthasarathy as an example of *failed* virtual diversity:

Virginia Tech professor and lead-in-water specialist Marc Edwards … concluded that Flint River water was so contaminated that it had leached lead from the pipes and caused the health problems. *Edwards was exactly the type of translator that [Collins, Evans, and Reyes-Galindo] envision*, and his intervention led Flint to switch its water supply back to the Detroit River. (our stress)

But Parthasarathy argues that Edwards’s specialist concentration on lead also prevented him appreciating other problems, such as Legionnaire’s disease, leading to the conclusion that virtual diversity must fail.

For this reason, Edwards could not have been ‘exactly the type of translator that we envision’: a more complete exponent of virtual diversity would have handled all the complaints one way or another. Edwards seems to have failed to accomplish what must be accomplished by successful ethnographers and anthropologists—an understanding of the society as a whole (Collins, 2020). When Parthasarathy and her colleagues are criticising Edwards, they see a half-empty glass in which virtual diversity has failed to address every aspect of the problem. In contrast, we see the same case study as a half-full glass in which virtual diversity has successfully resolved one aspect of the problem and shown, at least in principle, how other aspects could be addressed. As Thorpe may be intimating, those sociologists who consider that case studies should be based on sound understanding of the natural science in question are already trying to gain enough interactional expertise to manage virtual diversity. But we don’t know if Parthasarathy had this in mind and knew enough of the science to distinguish between local experts and committed stakeholders which is an essential element of virtual diversity (see Collins & Evans, 2002, pp. 249, 262).

Moving on to where we clearly differ:

(8) Our critics do not mention *VDiversity*’s argument that increasing diversity in science as a whole cannot provide epistemological gains except in unusual circumstances.
(9) *VCritique* ignores Harding’s idea of ‘strong objectivity’ which is one of *VDiversity*’s starting points, nor, Thorpe aside, is there any interest in the idea of an objective science strong, weak or middling. STS’s ‘Overton window’ has long closed on the possibility of scientific method being a useful warrant for knowledge.
(10) The crucial case studies in *VCritique* are local groups with experience-based expertise—‘local discrimination’ in the terms of Collins and Evans (2007, p. 14). Virtual diversity would give scientific integrity to the outcomes of these local controversies, preventing them from becoming matters of politics alone. It will also make the understanding of local controversies compatible with the universalistic aspirations of science that are vital if it is to act as a check and balance on populist dictators.

## The identity of science

Laurent stresses that science and politics are hard to separate. Collins (1975, 1981, p. 7, 1985) was one of the people who established this in the 1970s and ‘80s with the experimenter’s regress and the Empirical Program of Relativism. This work was done, however, in the spirit of what Laurent scorns as ‘dualism’.

In those early days we assumed there was science and there was politics. They could be as hard to separate as the water and oil in an emulsion, but they were still identifiable as separate substances (Collins et al., 2010). Laurent believes that to talk of science and politics as different is to make a ‘dualist’ mistake. But dualism is too abstract an idea to be *wrong* unless odd versus even is wrong, or wet versus dry is wrong, or rich versus poor is wrong.

Unfortunately, philosophers, along with STSers, seem to have given up on the ‘the problem of demarcation’. But demarcation lies at the heart of what Collins and Evans are trying to do, which is to use the idea of science as a defence against the erosion of truth in democracy. This turns not on a naturalistic description of the *profession*, or *occupation*, of science, but an analysis of what makes science distinctive as an institution even if many engaged in the *occupation* are not doing science as understood here. Given that politics and science are finely intermingled, we are also trying to reopen the Overton window and encourage STSers to think about science as a warrant for truth rather than a tool of oppression.^2^

Table 1 is a schematic description of science as an institution. The first column describes the institution’s *raison d’etre*, which is to discover correspondence truth— the truth about the observable world. This is the ‘formative intention’ or ‘formative aspiration’ (see Collins & Kusch, 1998, Ch. 2).

**Table 1.** Demarcating the institution of science.

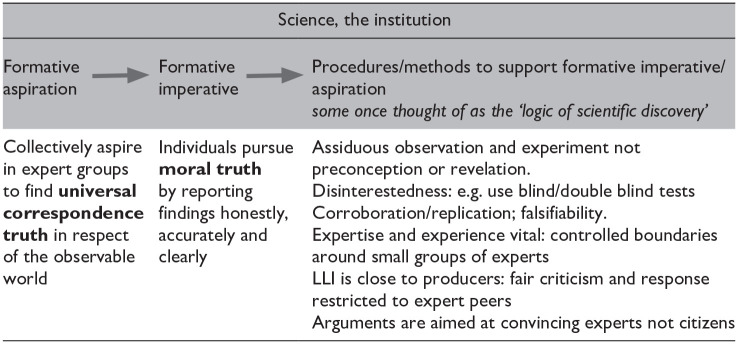					

The formative intention of science is pursued collectively and what follows in the second column is the ‘formative imperative’ for individuals—moral truth. Individuals must strive to report their contributions clearly and honestly if the collective goal is to have any chance of success.

An internal state like moral truth is easier to achieve than a collective aspiration but it is not a trivial matter. Scientists understand that expectancy effects, confirmation bias and many other ‘systematic errors’ vitiate moral truth and, in turn, confound correspondence truth. That is why, though the first two columns are necessary conditions for science, the third column is needed for sufficiency. The third column used to be thought of ‘scientific method’, or ‘the logic of scientific discovery’, but Wave 2 of Science Studies revealed that its elements were not logic-like rules but craft-like practices—the skills and values needed to realise the aspirations expressed in the first two columns.

*VCritique* sees column three differently. Parthasarathy says that virtual diversity would ‘[erase] the emotion, urgency, and complex understanding of problems and solutions that usually characterize the interventions of affected communities’; Subramaniam says the history of colonial oppression with which science is associated would alienate the public. And yet it is the attempt to set aside emotional engagement and historical commitments that make science what it is. Unless heated commitment to a hypothesis is refrigerated in column 3 it isn’t science that is being done. We recommend virtual diversity because the long apprenticeship needed to acquire the specific specialist knowledge and internalise the balance of the craft represented in the three columns is an unrealistic aim for most ordinary citizens. In science, including a scientific STS, commitment must be cooled.

### MMR and local discrimination

C&E’s ‘The Third Wave of Science Studies’ (2002), which inspired this exchange, was partly triggered by the 1990’s popular revolt against Measles, Mumps and Rubella vaccine (MMR). Colleagues in STS, led by Sheila Jasanoff and Brian Wynne, found themselves supporting those parents who felt convinced there was a link between MMR and autism in their children.^3^ Epidemiologists, on the other hand, knew that there was no detectable increase in the incidence of autism in countries that had adopted the MMR vaccine. To witness symptoms of autism in one’s child shortly after an MMR jab is emotionally overwhelming but statistically meaningless. Unfortunately, the emotion was exploited by a doctor (Andrew Wakefield) whose financial interests would later be revealed, along with his unethical behavior. Still more unfortunate, there is no emotional commitment to, and no local evidence pertinent to, the universalistic science of epidemiology.

The position of Collins and Evans (see e.g. Collins & Evans, 2017, pp. 80–83) that the correct response was to follow the epidemiological evidence and continue to use the MMR vaccine, was subject to attacks in which the argumentative style seemed more typical of the institution of law—a matter of representing a client and attacking the ‘opposition’—than contributing to the collective search for truth. The bottom line of Table 1’s third column indicates the difference between criticism that is intended to engage with other experts (including experience-based experts from local communities), which must start from a thorough and demonstrable comprehension of the adversary’s position, and criticism that is intended to persuade external audiences, including the general public and lay juries, which works better by simplifying and distorting the adversary’s position, or just ignoring it, thus sidestepping serious argument and simply *performing* one’s own view.^4^

The predictable outcome of the revolt against the MMR vaccine has now come to pass, with a widespread surge in measles epidemics risking the health and lives of children. But worryingly, for the truth-seeking credentials of our field, support from STS colleagues for the revolt has never been retracted nor have they publicly re-considered their work in the light of what has unfolded.^5^

### Local versus Universal

The Flint River revolt and the MMR revolt include both poles of the positions being argued here: The tensions are between the local versus the universal and the emotionally charged stakeholder versus the cooled down craft practice of science. That is the choice when we counterpose the science of child vaccination with political preferences of concerned parents and their supporters. The counter-emotional third column of Table 1, and the universalistic science of epidemiology championing an intangible future of herd immunity, will always struggle in a public sphere that is influenced by social media, disinformation, individual examples of local harms, a simplistic idea of individual freedom, and a readiness not to look up.^6^ Virtual diversity provides a way to redress this balance but requires cultural change from both the scientific community and the wider society.

## The bigger picture

Collins and Evans’s aspiration is to salvage truth in society by building from the institution of science. This is likely to be hard, impossible perhaps, but we can’t think of anywhere else to locate the foundations of the necessary change: In the US the politics of the ‘loyal opposition’ has been thoroughly defeated and the rule of law is being destroyed; big business, even that tasked with distributing and creating knowledge, is rolling over, and administrative experts are being replaced by political loyalists. In Putin’s Russia, the influential advisors are postmodernists, with a befuddled public allowing a leader in dialogue with Trump to define the truth as whatever is politically convenient. In contrast, like nearly all academics we are powerless if there is no respect for our craft practices. What was discovered under Wave Two wasn’t wrong and that is why we have to reconcile the discovery that there is experience-based expertise among non-scientists with the truth potential of a universalistic science. Virtual diversity can do this and will benefit both science and the public if it can be made to work. However unsophisticated this is, we want to say how things could be done better.

## Thorpe

Thorpe’s critique starts from the opponent’s position as scientific debate should. Thorpe has dug deep into the body of work that supports *VDiversity*, understanding how it relates to the concept of interactional expertise which in turn explains the division of labour and militates against identity politics. He also reminds us that as far back as 1972, Merton was concerned with ‘insiderism’ as an obstacle to universalism. He reflects on the relationship of our approach to Merton’s norms of science approach and recognises that ours starts from democracy and seeks support from science whereas Merton started from science and found support in democracy (Collins & Evans, 2017). If anyone wants to get a sense of the depth and consequence of the analysis of expertise that has grown out of the Third Wave, they could do worse than read the first 5 paragraphs of Thorpe’s critique. Reading on, they will find a deep understanding of what we are trying to accomplish with *VDiversity*.

When Thorpe gets to criticism, he turns to Wynne’s description of the interaction between Cumbrian sheep farmers and MAFF scientists. He cites a clumsy sentence of ours where what we should have said is that ‘there is almost certainly a marked asymmetry between the level of interactional expertise needed by the MAFF scientists and the sheep farmers to acquire a sufficient understanding of each other’s worlds’. Thorpe goes on to point out that Wynne, in a view like Subramaniam’s, said that the sheep farmers had a simmering resentment of the scientists because of ‘a large historical backlog of more private disbelief, mistrust, and alienation from the authorities’. We are uneasy about taking this on trust because of Wynne’s historic tendency to ‘represent clients’. For example, Wynne (1989, pp. 37–38) noted that some sheep farmers hosted MAFF scientists and learnt from them but, typically, there is no account of the scientists’ experience (and see footnote 9 of *VDiversity*). But taking Thorpe’s account at face value, we, along with Thorpe, Wynne and Subramaniam, regret the past overbearing behaviour of scientific authorities that has led to distrust of science and would like to change things. With a serious commitment to virtual diversity, scientists’ assiduous engagement with local groups could help even if, of itself, it would not resolve the more general problems of truth in society.

## Conclusion

*VDiversity* tries to find a way to bring about respect for local experienced-based expertise consistent with a universalistic science that can form a check-and-balance in democracy and a foundation for truth in society. Science and society are differently structured: *Science* aspires to be a set of *cognitively* bounded communities without geographical or demographic identity—core groups*. Societies*, in contrast, are spatially bounded and often demographically exclusive. In *VDiversity* we showed that a scientific profession that reflected the demographic diversity of the planet would hardly ever produce demographic representativeness in science’s core groups—it’s the arithmetic of superimposing very different social structures. One revealing exception is the balance of men and women when their ubiquitous expertises bear on the substance of the science. This is because, demographically, they are divided 50:50 throughout societies. We look here at the complement of that structural mismatch: Serious virtual diversity – which does not mistake advocacy for scientific debate – would enable experience-based experts to contribute to expert debates in a scientific manner, thereby improving the quality of the science and increasing the capacity of local communities to bring about change. That said, resolving problems in scattered and diverse social locales, even when it is done with scientific integrity, won’t bring about the change in civic culture needed to mend the relationship between society as a whole and science as a whole. For that, we all need to look up.

## Notes

1. The arguments found in *VDiversity* should be seen in the context of Collins and Evans (forthcoming): *Veritocracy: Truth, science, and how to preserve democracy*; the main thrust of *Veritocracy* can be quickly scanned in Collins (2023).

2. The 2002-onward move to the analysis of expertise and thence to the moral domain might be used to justify Latour’s (2004) sudden but philosophically unfounded conversion to the defence of climate-change science (strangely unmentioned in *VCritique*).

3. This observation is based on our own experience as participants in the debates. For Collins, a particularly significant moment occurred during the 2010 4S conference in Tokyo, during which he took part in a panel entitled ‘Beyond the “Third Wave”’, chaired by Trevor Pinch and featuring contributions from Brian Wynne, Steve Epstein, Frank Fischer and Miwao Matsumoto. For Evans, a similar encounter took place at a workshop on the theme of ‘Mixing things up: Science, politics and lay knowledge’ held at Lancaster University in March 2008. Evans’s notes from the meeting include clear statements from both Brian Wynne and Jerry Ravetz to the effect that parents expressing concerns about the safety of the MMR vaccine were asking ‘relevant questions’ that were being unreasonably dismissed. Published papers that take a similar view, and which were endorsed by many at the workshop, include Leach (2005) and Poltorak et al. (2005).

4. See Collins (2012). Oreskes and Conway (2010) document how these tactics have been used in a wide variety of science-related policy domains, whilst their more recent *Big Myth* (Oreskes & Conway, 2023) applies a similar perspective to the rise of neoliberal economics.

5. Jasanoff (2022) recently presented her views in a talk at Cambridge University, amongst other places (Democracy and distrust after the pandemic - Networks of evidence and expertise for public policy (cam.ac.uk)). Around 12 minutes in she explains that (our emphasis): ‘I think that even to begin to get to grips with the state we’re in, we have to begin by recognizing that these expert institutions that we’ve created are not simply telling us the state of the world in an unmediated fashion so that we can trust those claims in a direct way, instead they are exercising authority that is political *in the very same way* that our elected bodies are political, or our politically appointed bodies are political.’ Similar arguments, albeit not stated so clearly, can be found in publications such as Jasanoff (2022).

6. In the US, freedom of choice is a motif of anti-vax and at the time of the MMR revolt this was echoed by British Conservative MPs.
